# Transvaginal Pezzer Catheter Drainage in Treatment‐Resistant Deep Pelvic Abscess After Cesarean Section: Case Report

**DOI:** 10.1155/crog/7875567

**Published:** 2026-03-19

**Authors:** Bekir Kahveci, Eser Sefik Ozyurek

**Affiliations:** ^1^ Department of Obstetrics and Gynecology, Sanliurfa Training and Research Hospital, Sanliurfa, TR63330, Türkiye; ^2^ Department of Obstetrics and Gynecology, Bagcilar Training and Research Hospital, Istanbul, Türkiye, beh.gov.tr

**Keywords:** infection complication, pelvic abscess, Pezzer catheter, transvaginal drainage

## Abstract

A pelvic abscess occurs as an infectious complication of surgical intervention or as a result of infectious processes. Pelvic abscesses may be treated with broad‐spectrum antibiotics, which often fail, and surgical intervention is necessary. Surgical procedures include abscess drainage through laparotomy or laparoscopy. Alternative approaches include imaging‐guided abscess drainage in conjunction with antibiotics through transabdominal, transrectal, transvaginal or transgluteal routes. In this case, a Pezzer catheter was successfully applied to a deep pelvic abscess that was drained via the transvaginal approach. Transvaginal drainage with a Pezzer catheter following surgical drainage may be considered an effective treatment option for recurrent postoperative midline deep pelvic abscesses. Although this type of catheter is preferred by general surgeons, the advantage of using it vaginally by gynecologists offers an effective treatment approach. Additionally, it has a multifenestrated portion, which reduces the possibility of blockage during drainage.

## 1. Introduction

Pelvic abscess occurs as an infectious complication of surgery (e.g., hysterectomy, cesarean section [CS], and induced abortion) or the result of infectious processes (e.g., pelvic inflammatory disease, inflammatory bowel disease, and diverticulitis). Although pelvic abscesses after CS are rare and challenging to treat [1]. Postoperative abscesses present with vague symptoms, including nausea, vomiting, and sweating. Laboratory findings include leukocytosis with left shift, elevated erythrocyte sedimentation rate, and elevated C‐reactive protein (CRP) [2]. They are typically located in the paracolic grooves, pelvis, subdiaphragmatic areas, and between bowel loops, which are portions of the peritoneal cavity.

Although ultrasonography is highly sensitive for detecting superficial abscesses, its sensitivity for detecting deep pelvic or retroperitoneal abscesses is significantly lower than that of computed tomography (CT) and magnetic resonance imaging (MRI). For these reasons, CT or MRI, which is the gold standard for screening, is recommended in cases where there is an abscess clinic but no abscess can be detected on ultrasonography. Additionally, with advances in imaging modalities, drainage techniques (transabdominal, transrectal, transvaginal, and transgluteal approaches), and catheter technology, image‐guided pelvic abscess drainage has improved and become accepted as an effective alternative to surgery [3]. We herein report a case of deep pelvic abscess after CS treated with a Pezzer catheter.

## 2. Case Report

A 35‐year‐old patient (gravida, 4; para, 3) was admitted to our emergency department with abdominal pain, nausea, vomiting, and poor general condition on the 15th day after CS. Repeated CS was noted as the indication for surgery. On vaginal examination, there was malodorous discharge, and swabs were taken for culture. Cervical motion tenderness was positive. After the examination, a decision for relaparotomy was made with the preliminary diagnosis of intra‐abdominal abscess on CT.

During the operation, the abdomen was observed to be highly adherent, and the abscess was completely fenestrated and irrigated with saline. At the end of surgery, a catheter was placed in the abscess cavity. During follow‐up, blood CRP values increased following a short decremental period, and a CT was performed to rule out a recurrent intra‐abdominal abscess. When a 9 cm abscess area extending to the right paracolic area and bowel loops was detected on CT, a percutaneous drainage catheter was placed in the right paracolic area under radiology guidance. A total of 700 cc of purulent fluid was drained. This catheter was kept for 1 week, and intra‐abdominal washing was performed. CRP values increased following a transient fall. A CT scan was performed (Figure 1a). Since an image consistent with an abscess of ~10 cm in the pouch of Douglas was observed, placing a drainage catheter via a posterior approach was discussed with the interventional radiology department (Figure 1b).

Figure 1(a) Abscess appearance in Douglas on CT. (b) Vaginal ultrasonography showing an abscess in the Douglas. (c) Pezzer catheter (multifenestrated portion). (d) Transvaginal Pezzer catheter application from the posterior fornix.

**Figure:**
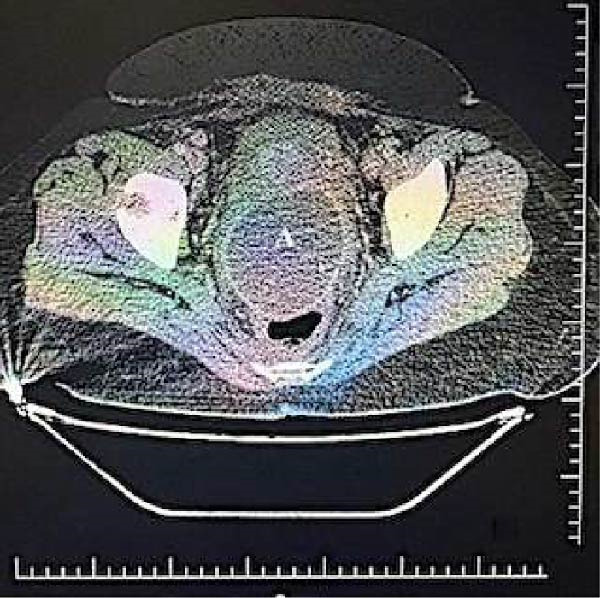
(a)

**Figure:**
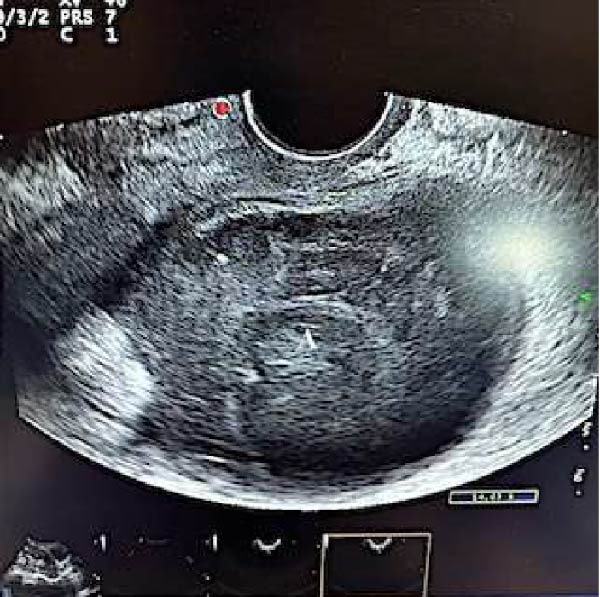
(b)

**Figure:**
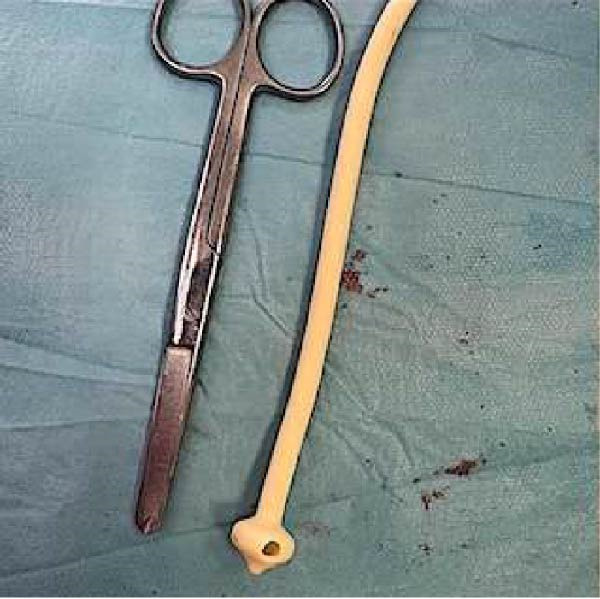
(c)

**Figure:**
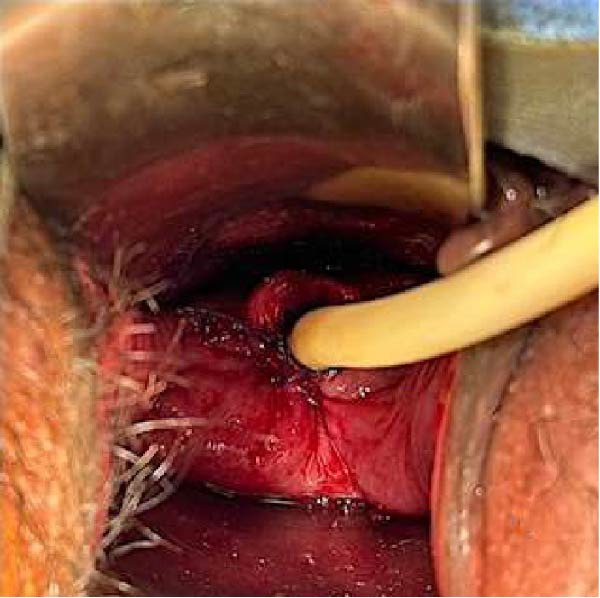
(d)

**Figure 2:**
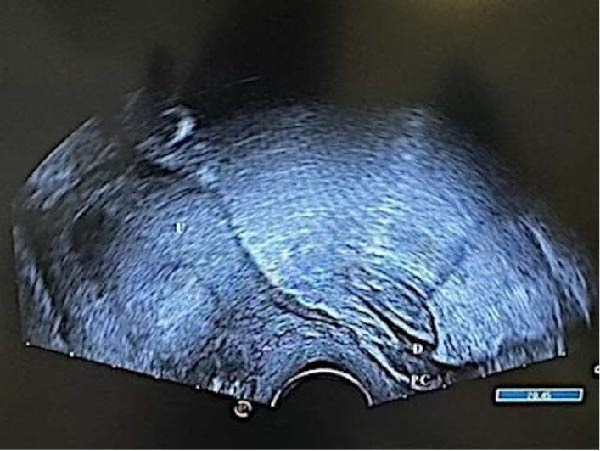
Douglas view on vaginal ultrasonography during Pezzer catheter removal.

We finally decided to perform a transvaginal drainage. A 1–2 cm transverse incision was performed transvaginally through the posterior fornix and a mushroom tip of the Pezzer catheter (fenestrated portion) (Figure 1c) was inserted through this incision into the pouche of Douglas as a minimally invasive approach (Figure 1d). The abdomen was washed with diluted hydrogen peroxide. The Pezzer catheter was kept for ~6 days, and it was withdrawn when clinical, laboratory, and radiologic improvements were observed, and the amount of daily drainage was less than 10 mL/24 h (Figure 2). The patient was discharged in good health condition as she felt clinically well, there was no increase in fever and infection parameters returned to normal levels. Informed consent was obtained from the patient for publication of this case report and accompanying images.

## 3. Discussion

Pelvic abscess formation is a rare complication of postpartum pelvic infections. We present a rare case of recurrent abscess formation following medical treatment and image‐guided drainage after CS involving the rectovaginal recess successfully treated with a transvaginal surgical intervention, pezzer catheter‐assisted drainage.

CS is currently the most common abdominal surgery procedure worldwide, and postcesarean infection usually occurs within the first 30 days after delivery [4]. A recent comprehensive study of 12,640 patients who underwent CS found that 23 of them (0.18%) developed postoperative pelvic abscesses [5]. When an intra‐abdominal abscess is detected, two basic management issues must be addressed. The first of these is “Does the abscess require drainage? and “What is the best drainage procedure? (percutaneous or surgical).” Second, in patients, “Should antibiotic treatment be continued or when should it be stopped?” Patients should be treated empirically with broad‐spectrum parenteral antibiotic regimens that have both aerobic and anaerobic activity [6]. Image‐guided pelvic abscess drainage and antibiotic support can reduce abdominal sepsis and complication rates, improve the general condition (clinical success rates were 93.9%), and prevent early surgery in patients [7]. In a study of 81 surgical patients whose postoperative abscesses were treated with percutaneous drainage and antibiotics, 22% still required subsequent surgery [8]. In this case, it is considerable that the management of recurrent abscess after surgery is complicated and the process is long. On the other hand, another study shows that laparoscopic drainage of postcesarean pelvic abscesses may be an effective approach in cases where image‐guided drainage is impracticable [1].

According to recent studies, for patients with abscesses 7 cm or larger, we perform abscess drainage in conjunction with parenteral broad‐spectrum antibiotics. Although abscesses smaller than 7 cm may respond to antibiotics alone, percutaneous drainage with parenteral antibiotic therapy results in rapid resolution. Therefore, for initial treatment of abscesses <7 cm but larger than 2 cm, we recommend empirical antibiotics and percutaneous drainage. Patients with abscesses 2 cm or smaller usually respond to antibiotic therapy alone [9]. Surgery is technically challenging because of anatomical distortion, extensive adhesions, necrosis, and inflammation that can cause thickening of the peritoneum. Therefore, percutaneous drainage of intra‐abdominal abscesses is a safe procedure with low complication rates. In deep pelvic abscesses, safe percutaneous intervention is difficult because of the involvement of multiple organs and structures. When the anterior approach is not possible due to these obstacles to percutaneous drainage, the transgluteal approach, transvaginal approach and transrectal approach are recommended. When minor complications were evaluated, it was seen that the catheter‐related minor complication rates were 3.9%, 8.3%, 10%, and 13.3% for transabdominal, transvaginal, transrectal and transgluteal access routes, respectively [7]. But in the present case, due to the difficulty of the anterior approach, the Pezzer catheter drainage method, which is preferred by general surgeons rather than gynecology practice was preferred. The catheter was placed in the Douglas via the transvaginal route. The purpose of using a Pezzer catheter is to provide adequate drainage to prevent drain blockage during the healing process [10].

In conclusion, transvaginal drainage with a Pezzer catheter following surgical drainage may be considered an effective treatment option for recurrent postoperative midline deep pelvic abscesses after CS. Although this type of catheter is preferred by general surgeons, the advantage of using it vaginally in gynecologists offers an effective treatment approach. Additionally, it has a multifenestrated portion, which reduces the possibility of blockage during drainage

## Author Contributions

Conceptualization, methodology, funding acquisition, resources: Bekir Kahveci. Formal analysis, investigation, writing – original draft preparation, writing – review and editing, supervision: Bekir Kahveci and Eser Sefik Ozyurek.

## Funding

This study had no funding.

## Consent

Informed consent was obtained from the patient for publication of this case report and accompanying images.

## Conflicts of Interest

The authors declare no conflicts of interest.

## Data Availability

The data that support the findings of this study are available in the supporting information of this article.
